# Msp40 effector of root-knot nematode manipulates plant immunity to facilitate parasitism

**DOI:** 10.1038/srep19443

**Published:** 2016-01-22

**Authors:** Junhai Niu, Pei Liu, Qian Liu, Changlong Chen, Quanxin Guo, Junmei Yin, Guangsui Yang, Heng Jian

**Affiliations:** 1 Department of Plant Pathology and Key Laboratory of Plant Pathology of Ministry of Agriculture, China Agricultural University, Beijing 100193, P. R. China; 2 Tropical Crops Genetic Resources Institute, CATAS/Key Laboratory of Crop Gene Resources and Germplasm Enhancement in Southern China, Danzhou, Hainan 571737, P. R. China

## Abstract

Root-knot nematodes (RKNs) are obligate biotrophic parasites that invade plant roots and engage in prolonged and intimate relationships with their hosts. Nematode secretions, some of which have immunosuppressing activity, play essential roles in successful parasitism; however, their mechanisms of action remain largely unknown. Here, we show that the RKN-specific gene *MiMsp40*, cloned from *Meloidogyne incognita*, is expressed exclusively in subventral oesophageal gland cells and is strongly upregulated during early parasitic stages. *Arabidopsis* plants overexpressing *MiMsp40* were more susceptible to nematode infection than were wild type plants. Conversely, the host-derived *MiMsp40* RNAi suppressed nematode parasitism and/or reproduction. Moreover, overexpression of *MiMsp40* in plants suppressed the deposition of callose and the expression of marker genes for bacterial elicitor elf18-triggered immunity. Transient expression of *MiMsp40* prevented Bax-triggered defence-related programmed cell death. Co-agroinfiltration assays indicated that MiMsp40 also suppressed macroscopic cell death triggered by MAPK cascades or by the ETI cognate elicitors R3a/Avr3a. Together, these results demonstrate that MiMsp40 is a novel *Meloidogyne*-specific effector that is injected into plant cells by early parasitic stages of the nematode and that plays a role in suppressing PTI and/or ETI signals to facilitate RKN parasitism.

Root-knot nematodes (RKNs; *Meloidogyne* spp.) are among the most devastating plant-parasitic nematodes (PPNs). These specialized sedentary endoparasites attack more than 3,000 plant species and cause up to 5% losses in global crop yield^1^. Infective second-stage juveniles (J2s) completely penetrate plant roots and settle near the vascular tissues, where they induce the formation of elaborate giant cells (GCs) that serve as their permanent feeding sites. The nematode dwells adjacent to the giant cells, where females develop until they eventually achieve reproductive maturity. Secretions produced by nematode amphids, hypodermis, and oesophageal glands (dorsal and subventral) are involved in complicated nematode-plant molecular interactions during the parasitism process; these oesophageal gland cells synthesize protein effectors secreted through the nematode stylet into plant cells, where they play essential roles in parasitism initiation and maintenance.

During the past two decades, multiple candidate effector genes have been isolated by diverse strategies such as cDNA-AFLP^2^, cDNA library screening^3^,^4^, proteomic analysis^5^,^6^, transcriptome and genome sequencing^7^,^8^,^9^,^10^, and bioinformatic mining^11^,^12^,^13^. Although only a small percentage of parasitism genes have been functionally elucidated in detail, advances in the identification and functional analysis of phytonematode effectors have greatly expanded our understanding of the unique host-parasite interactions and begun to clarify the regulation of the parasitic process by the nematode^14^,^15^,^16^,^17^,^18^. Based on the primary functions that benefit the parasite, these secretions can be roughly divided into the following groups: (I) chemotaxis mediators involved in locating hosts and suitable feeding sites; (II) cell wall degrading and modifying enzymes to assist with the penetration and migration of the nematode into and within roots; (III) plant reprogramming regulators involved in the formation and maintenance of feeding cells; (IV) putative modulators of host metabolism for the production of nematode-required nutrients; and (V) immunomodulatory components for protecting nematodes against plant defence responses or shielding feeding cells from localized programmed cell death (PCD). Among these molecules, immunosuppressors have been postulated to play decisive roles because successful parasitism is heavily reliant on whether nematodes and feeding cells elude or survive attack by the plant immune system^15^,^16^,^17^.

Currently, the evolution of inducible defence responses in plant-pathogen interactions is considered to follow the ‘zig-zag’ model^19^. In phase 1 of this model, pathogen-associated molecular patterns (PAMPs) are recognized by transmembrane pattern recognition receptors (PRRs), resulting in PAMP-triggered immunity (PTI) that halts further ingression of most phytopathogens. In phase 2, adapted pathogens secrete effector proteins to evade and/or suppress PTI, resulting in effector-triggered susceptibility. In phase 3, a given effector is specifically recognized (indirectly or directly) by intracellular resistance (R) gene receptors, resulting in effector-triggered immunity (ETI), which is usually associated with disease resistance and a hypersensitive cell death response (HR) at the infection site. Although no secretion has been definitely established as a PAMP in PPNs, several lines of evidence indicate that PPNs have effectors that can modulate PTI or ETI during nematode-plant interactions. Microarray and transcriptome analyses of infected root tissues and feeding cells have elucidated plant defence suppression; for example, many defence-related genes are downregulated during plant-nematode interactions^7^,^20^,^21^,^22^. *Heterodera schachtii* and *M. javanica* infections activate *Arabidopsis* 13-lipoxygenase *LOX4*, whereas functionally deficient *LOX4* plants have marked increases in the numbers of females and eggs or in the female/male ratio, accompanied by the accumulation of jasmonic acid and the increased expression of allene oxide synthase, allene oxide cyclase, and ethylene-responsive transcription factor 4. These results indicate that the nematode modifies *LOX4*-mediated plant defence^23^. In another recent report, *H. schachtii* was shown to activate the NADPH oxidases *RbohD* and *RbohF*, thereby producing reactive oxygen species (ROS). In this manner, the nematode may fine tune the pattern of plant cell death during root invasion, potentially antagonizing salicylic acid-induced defence responses^24^.

Selected effectors with defence suppression activity have been characterized in various PPN species. In *H. schachtii*, the *Hs10A06* effector targets *Arabidopsis* spermidine synthase and causes an increase in antioxidant protection and an interruption of salicylic acid signalling in the syncytia, suggesting that *Hs10A06* modulates host defence responses^25^. The *H. schachtii* annexin-like effector *4F01* interacts with an *Arabidopsis* oxidoreductase of the *2OG-Fe* (II) oxygenase family, an enzyme that promotes susceptibility to pathogens^26^. Another *annexin*-like gene from *H. avenae* can suppress Bax-triggered PCD (BT-PCD), targeting the two downstream kinases MKK1 and NPK1 in the mitogen-activated protein kinase (MAPK) signalling pathway^27^. The *H. schachtii 30C02* effector can interact with β-1,3-endoglucanase, a potential PR protein, and may thus be involved in defence suppression^28^. In *Globodera* species, *G. rostochiensis* SPRYSEC-19 binds directly to a CC-NB-LRR type of the disease resistance protein SW5-F, and this association has been demonstrated to suppress the ETI response^29^. In *Nicotiana benthamiana* leaves, the transient expression of *GrCEP12*, an ubiquitin carboxyl extension protein secreted by *G. rostochiensis* dorsal gland cells, suppresses the production of ROS and the induction of PTI marker genes triggered by the bacterial PAMP flg22. These results provide direct evidence that GrCEP12 is involved in the suppression of PTI^30^. *GrVAP1*, a *G. rostochiensis* venom allergen protein expressed in the subventral gland cells, specifically affects PCD mediated by surface-localized immune receptors, suppressing host defences activated by host detection of plant cell wall fragments released by migrating nematodes during the onset of parasitism^31^. In *Meloidogyne* species, near-isogenic nematode populations have been shown to be avirulent or virulent to plants carrying the RKN resistance gene *Mi*, and the amphid-secreted protein MAP-1 seems to be involved in *Mi*-related ETI^32^. Transgenic *Arabidopsis* plants overexpressing *Mi-CRT* (*M. incognita* calreticulin) show increased susceptibility to *M. incognita*. The induction of defence genes and the deposition of callose were suppressed after treatment of these plants with the PAMP elf18, demonstrating that Mi-CRT has a direct effect on PTI suppression^33^. Although understanding of the above immunomodulatory components has progressed, additional related effectors in PPNs may remain unidentified because of the complexity of the nematode-plant interaction and because numerous effectors with defence suppression activity occur in other plant pathogens (including bacteria^34^, fungi^35^,^36^ and oomycetes^37^).

In the present study, we report the functional characterization of the *M. incognita* putative oesophageal gland cell secretory protein^40^ (*MiMsp40*) using an integrative approach. Our results show that *MiMsp40* was strongly upregulated during the early parasitic stages. Plants overproducing *MiMsp40* were more susceptible to *M. incognita*. Conversely, host-derived RNAi of *MiMsp40* decreased the ability of nematodes to infect the plants. Moreover, transient expression of *MiMsp40* by agroinfiltration in *N. benthamiana* leaves prevented BT-PCD, and constitutive expression of *MiMsp40* suppressed PTI triggered by PAMP elf18 in *Arabidopsis thaliana*. Further transient co-expression showed that MiMsp40 suppressed cell death induced by NPK1, MKK1 and R3a (with its cognate elicitor AvrR3a from *Phytophthora infestans*) but not by Gpa2 (with its cognate elicitor RBP1 from *G. rostochiensis*). Considered together, our results suggest that *MiMsp40* represents a novel *Meloidogyne* immunomodulatory effector that suppresses the activation of plant cell death associated with PTI and/or ETI responses and thereby promotes nematode parasitism.

## Results

### Analysis of the *MiMsp40* sequence and genome distribution

**Figure 1:**
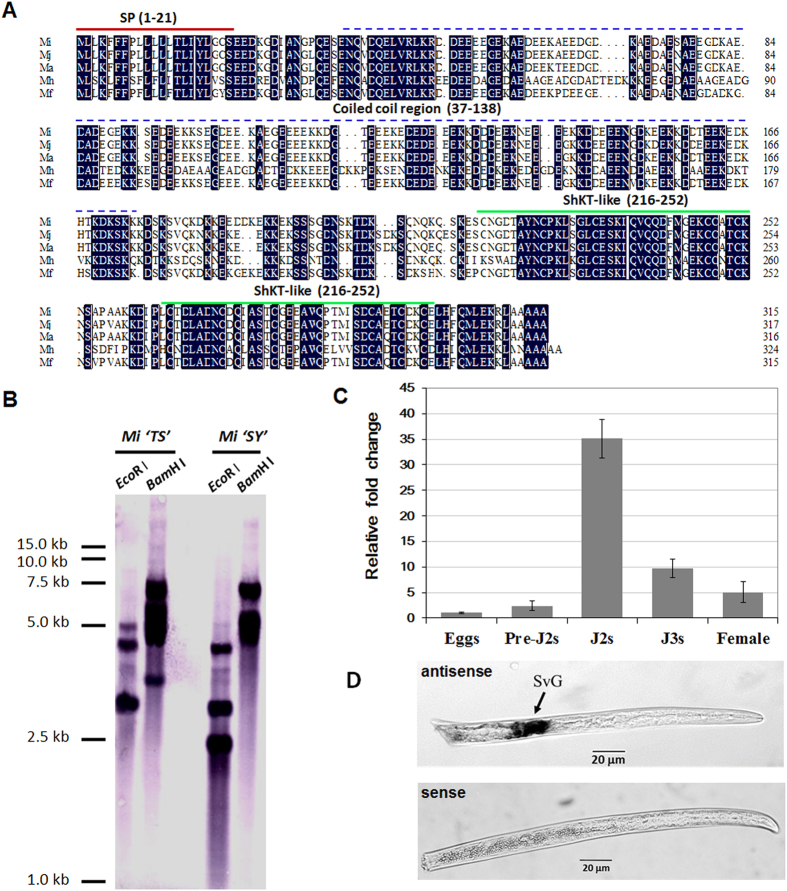
Characterization of *M. incognita Msp40*. (**A**) Multiple sequence alignment of the predicted *Meloidogyne* Msp40-like protein sequences. Absolute conserved residues are shaded dark. The underlined sequences indicate predicted signal peptides (1–21 aa), the *coiled*-*coil* region (37–174 aa) and two ShKT motifs (216–252 aa, 264–300 aa). (**B**) Southern blotting to determine the genomic copy number of *MiMsp40* for *M. incognita* strains ‘TS’ and ’SY’ revealed that *MiMsp40* belongs to a small gene family composed of at least three members. (**C**) Developmental expression pattern of *MiMsp40*. The relative mRNA expression level of *MiMsp40* was quantified by qPCR in five different *M. incognita* life stages: eggs, pre-J2s, par-J2s, J3s and females. (**D**) *In situ* localization of *MiMsp40* mRNA. Digoxigenin-labelled antisense *MiMsp40* cDNA probe (dark staining) to transcripts specifically expressed within the subventral pharyngeal glands (SVG) of a parasitic juvenile. scale bar = 20 μm.

An 1150 bp *MiMsp40* cDNA sequence containing an open reading frame of 948 bp was isolated by RT-PCR using RNA extracted from mixed parasitic stages of *M. incognita*. This sequence encoded a 315 amino acid protein with an N-terminal signal peptide (SP, 1–21 aa) for secretion, a coiled-coil region (37–174 aa), and two 37-aa putative C-terminal ShK toxin (ShKT) structures (216–252 aa, 264–300 aa) (Fig. 1A). The cleavage site between the 21 and 22 aa positions indicates that MiMsp40 may be targeted for secretion outside the nematode gland cells and may enter plant cells, a typical characteristic of phytoparasitic nematode effector proteins. A DIG-labelled probe derived from *MiMsp40* was hybridized in a DNA gel blot to *Eco*RI- and *Bam*HI-digested genomic DNA from *M. incognita* strains ‘TS’ and ‘SY’. Three or four DNA-hybridized bands ranged from 2 to 10 kb in the *M. incognita* genome, revealing that *MiMsp40* belongs to a small gene family composed of at least three analogues (Fig. 1B). This finding is consistent with the BLAST analysis of the *M. incognita* genome database, which revealed three homologous genes.

### *MiMsp40* orthologue identification and phylogenetic analysis

*M. arenaria* and *M. javanica* orthologues to the cDNA and genomic DNA were cloned using a homology-based PCR approach. *M. hapla* (Mh10g200708_Contig1732) and *M. floridensis Msp40*-like genes (M_floridensis_ nMf.1.0.scaf04684) were obtained through a BLAST analysis of their assembled genome sequences, and the predicted coding cDNA and protein sequences are presented in Figs 1A and S1A. All the *Msp40*-like genes had six exons and five introns with similar intron/exon distributions (Fig. S1A). One subtle distinction is that *MfMsp40* (1314 bp) was longer than the other genes (1233–1245 bp) because of the significantly larger second intron. The MiMsp40 protein shared 51.4–95.0% sequence identity with the other four *Meloidogyne* species homologues (Fig. S1B). A consensus tree based on the analysis of Msp40-like protein sequences divided the five tested RKN genes into three clades agreeing with phylogenetic relationships previously developed on the basis of morphology, reproduction mode, karyotype and other molecular characteristics (Fig. S1B)^38^,^39^. Although we screened a large amount of released genomic data from plant-parasitic nematodes, including the soybean cyst nematode *H. glycines*, the potato cyst nematodes *G. rostochiensis* and *G. pallida*, and the pinewood nematode *Bursaphelenchus xylophilus*, as well as bioinformatics resources for other plant-parasitic, animal-parasitic and free-living nematodes (Table S1), we found no significant database matches other than the *Meloidogyne* species. Therefore, this analysis classified *Msp40* as a novel *Meloidogyne* genus-specific protein-coding gene.

### Developmental and spatial expression of *MiMsp40* within *M. incognita*

The developmental expression of *MiMsp40* during the egg, hatched pre-parasitic J2 (pre-J2), parasitic J2 (par-J2), J3 and J4 stages was quantified by qRT-PCR. Compared to the egg stage, the mRNA levels found during the four later stages were 2.4-, 35.2-, 9.7- and 5.0-fold higher (Fig. 1C). The maximum level of mRNA expression was detected in parasitic J2s and the level decreased in later sedentary stages, suggesting a primary role of *MiMsp40* during the early stages of nematode parasitism. *In situ* hybridization demonstrated that the *MiMsp40* mRNA was locally expressed specifically within the subventral oesophageal gland secretory cells of parasitic stage J2s (Fig. 1D), no signal was detected when control sense cDNA probes were used, consistent with previous results^4^.

### Subcellular localization of MiMsp40 in plant cells

In experiments to identify the subcellular localization of *MiMsp40* (SP+): *eGFP* and *MiMsp40* (SP−): *eGFP* constructs under the control of the CaMV 35 S promoter, the eGFP signals were localized to the cytoplasm and nuclei of transformed onion cells. No specific pattern suggestive of organelle or membrane localization was found in these cells. The only noticeable pattern was that the signals occurred within the transformed plant cells rather than outside the cells (Fig. S2). These data were consistent with the prediction of cytoplasmic localization (cytoplasm, certainty = 0.65) using Psort software, suggesting that MiMsp40 may function in the interior of host cells. Moreover, the constructs coincided with the *H. schachtii* CBP effector^40^, confirming that the MiMsp40 signal peptide did not function properly in *planta*.

### Constitutive expression of *MiMsp40* in *Arabidopsis* resulted in altered morphology and susceptibility to *M. incognita*

**Figure 2:**
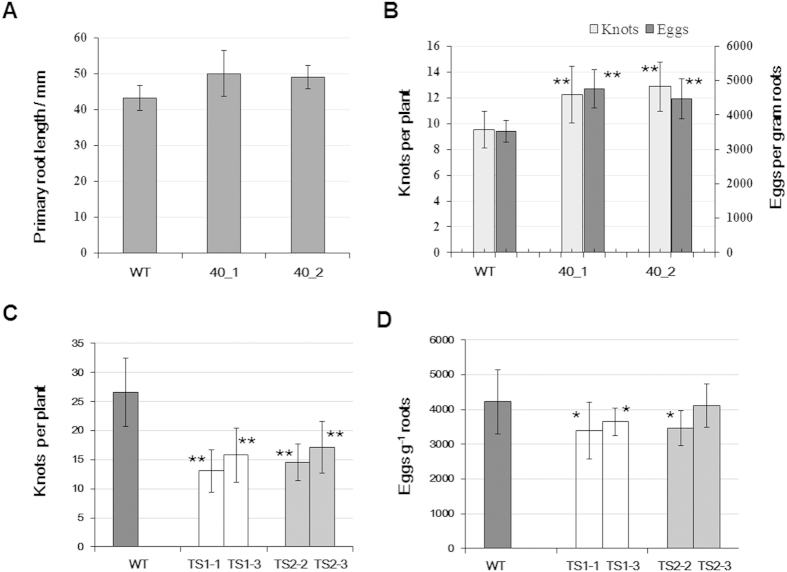
Effect of ectopic expression and host-derived RNAi of *MiMsp40* on the phenotype and nematode susceptibility of the plant. *Arabidopsis MiMsp40* expression homozygous T3 lines accelerated the root growth (**A**) and the numbers of knots/eggs (**B**) compared to WT lines, whereas the RNAi lines developed relatively fewer knots (**C**) and eggs (**D**) compared to the WT line. The parasitic females laid fewer eggs. Each bar value represents the mean ± SD of n > 25. “*” or “**” indicates significant differences based on Student’s *t*-test (*P* < 0.05 or *P* < 0.01).

We determined whether *MiMsp40* overexpression produces parasitism-associated phenotypes such as changes in plant growth and/or nematode susceptibility similar to those produced by selected identified PPN effectors such as *Hg-SYV46*^41^, *Mi16D10*^42^, *HsCBP1*^40^, *Hs30C02*^28^, and *GrUBCEP12*^30^. Multiple independent homozygous *Arabidopsis* lines overexpressing *MiMsp40* were generated through transformation and continuous self-crossing, and the lines selected for RKN infection were based on the *MiMsp40* expression levels as measured by RT-PCR analysis. Seeds from the six chosen homozygous lines were planted in MS medium, and the root length was measured 14 d after planting. The *MiMsp40* lines produced plants with significantly increased root lengths ranging from 49.1 ± 7.56 mm to 50.0 ± 6.44 mm compared with the wild-type (WT, Col-0) control (44.3 ± 3.44 mm), and the average increase was 10.7–13.1% (Fig. 2A). The morphological changes demonstrate that *MiMsp40* has a biological function in plants.

To investigate the effect of *MiMsp40* ectopic expression on parasitic susceptibility, *Arabidopsis* WT and *MiMsp40* roots were inoculated with *M. incognita* J2s. Six weeks after infection, the numbers of galls and eggs were counted in each plate. The *MiMsp40* roots displayed significant increases in both the number of galls and the number of eggs per gram root (*P* < 0.05, Fig. 2B), with average rates of increase of 28.1–35.0% and 27.1–35.1%, respectively. The galls of *MiMsp40* root systems were also larger than those of WT root systems. All infection tests on each transgenic line were performed using two independent biological experiments to determine consistency of results.

### Effect of host-derived RNAi of *MiMsp40* on root infection by *M. incognita*

To further determine the function of *MiMsp40* in successful parasitism, two RNAi structures targeting *MiMsp40* 5′ and 3′ ORF sequences were developed and transferred into *Arabidopsis*, and homozygous lines were used for nematode challenge assays. At 7 weeks post-inoculation, *Arabidopsis* lines possessing RNAi constructs and control WT lines were analysed to determine changes in nematode susceptibility. By monitoring nematode development, we found that nematodes in both RNAi harbouring and control WT roots reached the mature female stage but that RNAi roots developed significantly fewer root galls (*P* < 0.01, Figs 2C, S3). For two TS1 RNAi lines (TS1-1, TS1-3), the number of galls decreased by 50.9% and 40.5%, respectively, while for TS2 RNAi lines (TS2-2, TS2-3), the rates of decrease were 45.1% and 35.4%, respectively (Fig. 2C). In contrast, the number of eggs from RNAi-treated roots showed only a limited decrease compared with those from WT roots, and the TS2-2 line did not display a statistically significant difference compared with wild type (*P* > 0.05, Fig. 2D). For the TS1-1, TS1-3 and TS2-2 lines, the egg masses g^−1^ root were 17.8%, 13.8% and 18.3% reduced, respectively. The results indicate that host-derived RNAi of *MiMsp40* impairs *M. incognita* parasitism and/or propagation, and the combined data provide empirical support that *MiMsp40* is an essential parasitism gene for *M. incognita* infection of plants.

### MiMsp40 suppresses immune-associated PCD in *N. benthamiana*

To extend our understanding of the function of *MiMsp40*, we investigated the possible role of *MiMsp40* in suppressing host defences during parasitism.

**Figure 3:**
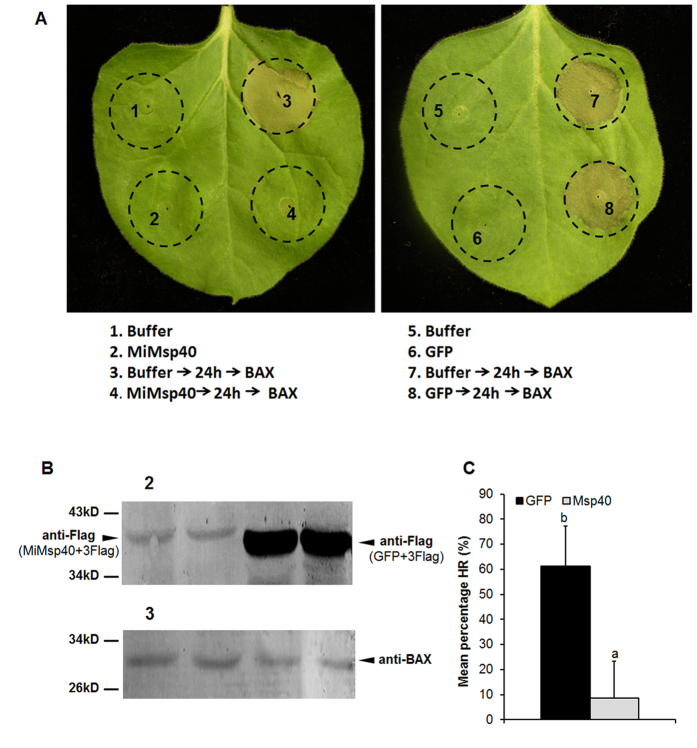
MiMsp40 suppresses BT-PCD. (**A**) MiMsp40 suppresses BT-PCD. *N. benthamiana* leaves were infiltrated with buffer or *Agrobacterium* cells carrying the *MiMsp40* gene or the negative control *GFP* gene, either alone or 24 h before infiltration with *Agrobacterium* cells carrying the mouse *Bax* gene. Representative images were acquired 5 d after the last infiltration. (**B**) Western blot analysis of the expression of MiMsp40, GFP and BAX. (**C**) The average areas of PCD lesion of MiMsp40 and control GFP followed by BAX expression. Each column represents the mean with standard deviation (n = 15). ‘a’, statistically significant difference from GFP controls; ‘b’, no statistical difference from controls by Student’s *t*-test (*P* < 0.01).

*MiMsp40* consistently suppressed BT-PCD when infiltrated 24 h before *Bax* (the average area of PCD lesions was 9%), whereas infiltrating with *Bax* alone initiated a typical BT-PCD reaction. Infiltration with *Agrobacterium tumefaciens* strain GV3101 cells carrying a green fluorescent protein gene (*GFP*) instead of *MiMsp40* did not suppress BT-PCD (the average area of PCD lesions was 61% (Fig. 3A,C). The accumulation of MiMsp40 and GFP proteins in infiltrated tissues was confirmed by immunoblotting using anti-Flag because of their fusion with the 3× Flag tag peptide (Fig. 3B). Bax accumulation in tissues was also validated by immunoblots, eliminating the possibility that the suppression of BT-PCD resulted from the breakdown of Bax synthesis.

**Figure 4:**
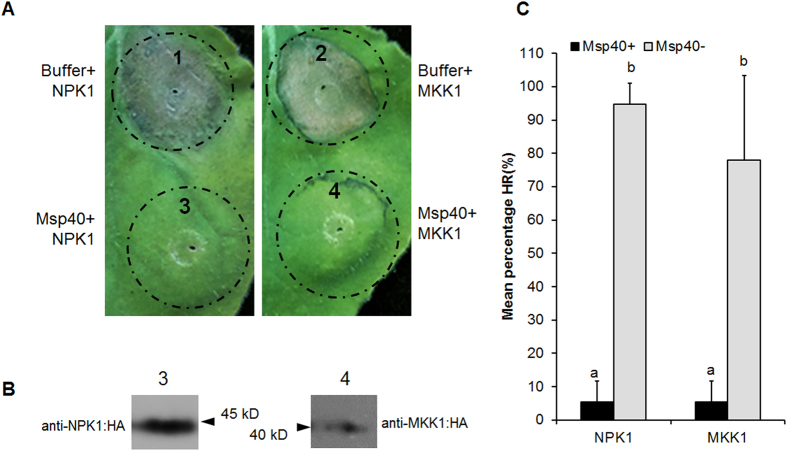
MiMsp40 suppresses MAPK cascade-associated cell death. (**A**) MiMsp40 suppresses MKK1- or NPK1^Nt^-triggered cell death. *N. benthamiana* leaves were infiltrated with buffer or *Agrobacterium* cells carrying the *MiMsp40* gene 24 h before infiltration with *Agrobacterium* cells carrying the *MKK1* or *NPK1*^*Nt*^ gene. Representative images were acquired 5 d after the last infiltration. (**B**) Western blot analysis of the expression of MKK1 and NPK1^Nt^. (**C**) The average areas of PCD lesion of MiMsp40 and buffer control followed by MKK1 or NPK1^Nt^ expression. Each column represents the mean with standard deviation (n = 15). ‘a’, statistically significant difference from controls; ‘b’, no statistical difference from controls by Student’s *t*-test (*P* < 0.01).

Plant MAPK cascades consist of three protein kinases. MAPKKKs (MAPKK kinases) regulate MAPKKs (MAPK kinases) through phosphorylation. In turn, MAPKKs regulate MAPK through phosphorylation. These kinases are key players and function in concert in both the PTI and ETI signalling pathways. To examine the effect of MiMsp40 on MAPK cascade-associated cell death, we tested the suppression of PCD triggered by genes encoding MKK1 (a MAPK kinase) or by the N terminus of NPK1 (residues 1 to 373; NPK1^Nt^) (a MAPKK kinase) when introduced into *N. benthamiana* by agroinfiltration. The results of thrice-repeated experiments (with 15 leaves each) showed that cell death around agroinfiltrated sites was strongly induced by MKK1 or NPK1^Nt^ alone. In contrast, sites at which MiMsp40 infiltration was followed by infiltration of MKK1 or NPK1^Nt^ displayed considerably less necrosis (Fig. 4A). Quantitatively, the average PCD lesion area for co-agroinfiltration (5%) was obviously smaller than those of MKK1 (78%) and NPK1^Nt^ (95%) (*P* < 0.05, Fig. 4C). The protein levels of MKK1 and NPK1^Nt^ in the presence of MiMsp40 were similar in the immunoblots (Fig. 4B), indicating that this inhibition is not achieved through an effect on protein expression or stability. These assays reveal that MiMsp40 is a potent suppressor of macroscopic cell death triggered by MAPK cascades and specifically targets processes downstream of MKK1 and NPK1.

**Figure 5:**
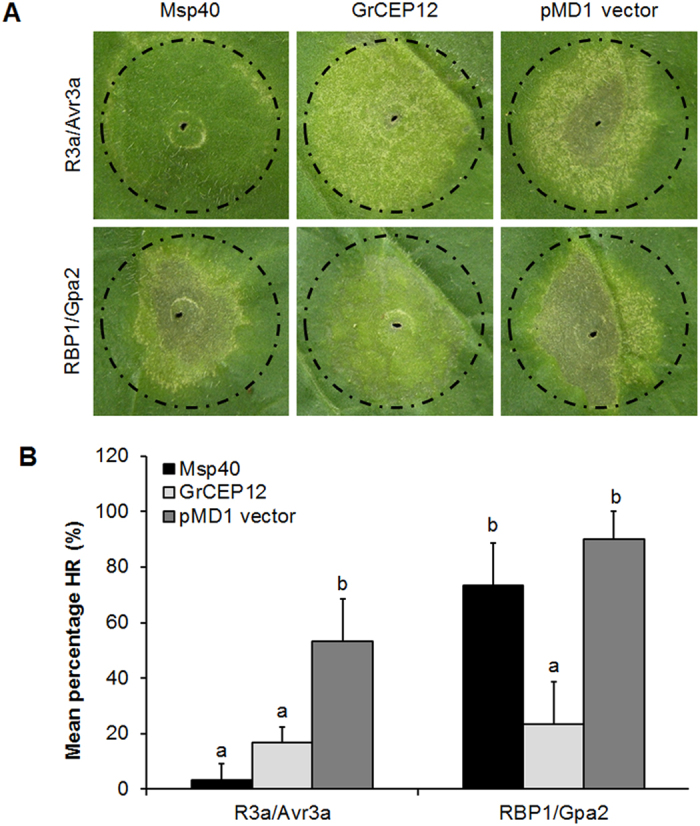
MiMsp40 suppresses R3a/Avr3a (but not Gpa2/RBP-1)-triggered cell death. (**A**) Graph showing the necrosis triggered by co-infiltration. *N. benthamiana* leaves were infiltrated with *Agrobacterium* cells carrying the indicated constructs 24 h before infiltration with *Agrobacterium* cells carrying *R3a/Avr3a* or *Gpa2/RBP-1*. The empty pMD1 vector was used as a negative control. GrCEP12 was employed as a positive control for its ability to suppress cell death triggered by both combinations. (**B**) Average areas of PCD lesion of co-infiltration sites. Each column represents the mean with standard deviation (n = 15). ‘a’, statistically significant difference from controls; ‘b’, no statistical difference from controls by Student’s *t*-test (*P* < 0.05 or *P* < 0.01, respectively).

To further evaluate a role for MiMsp40 in suppressing cell death associated with the ETI response, *A. tumefaciens* strain GV3101 cells carrying constructs of pMD1:MiMsp40, negative control (empty vector pMD1) and positive control (pMD1:GrCEP12) were infiltrated into *N. benthamiana* leaves 24 h before infiltration of *A. tumefaciens* cells carrying R3a/Avr3a or Gpa2/RBP-1. As expected, pMD1 did not affect the hypersensitive response induced by either R3a/Avr3a or Gpa2/RBP-1 (the average areas of PCD lesions were 53.3% and 90%, Fig. 5), whereas GrCEP12 suppressed cell death triggered by both groups of cognate elicitors (the average areas of PCD lesions were 16.7% and 23.3%, Fig. 5), as previously reported^30^. In contrast, MiMsp40 suppressed cell death induced by R3a/Avr3a but not by Gpa2/RBP-1 (Fig. 5A). Significant differences in the mean percentage of HR were observed between the MiMsp40 co-agroinfiltrated sites and the negative controls (the average areas of PCD lesion were 3.3% and 73.3%, Fig. 5B). Therefore, *MiMsp40* may represent a novel immunomodulatory effector that suppresses the activation of plant cell death associated with both PTI and ETI responses.

### *MiMsp40* overexpression suppresses *Arabidopsis* callose deposition induced by elf18

**Figure 6:**
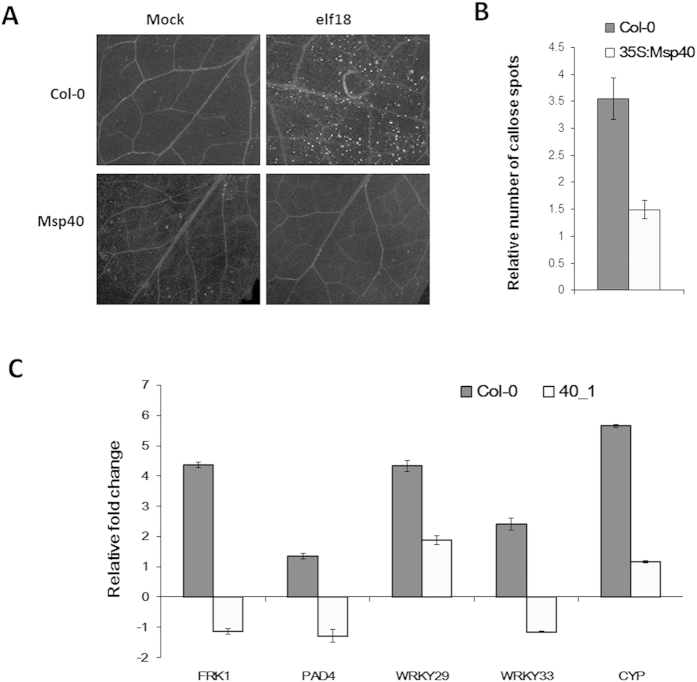
MiMsp40 suppresses callose deposition triggered by elf18. *MiMsp40* transgenic and WT (Col-0) *Arabidopsis* plants were inoculated with 1 μM elf18. Callose (white dots) was visualized (**A**) and quantified (**B**) 24 h after infiltration by staining with aniline blue. (**C**) Expression of plant defence genes. The mRNA expression levels of *FRK1*, *PAD4*, *WRKY33*, *WRKY29* and *CYP81F2* were measured by qPCR in *MiMsp40* transgenic and WT plants treated with 1 μM elf18. *Arabidopsis UBP22* was used as an internal control to normalize the gene expression level. Changes in gene expression were calculated using the 2^−△△CT^ method; the data are presented as averages of three independent biological experiments, each consisting of three technical replicates.

Callose deposition is a well-studied component of the *Arabidopsis* PTI response at the site of infection^43^. We tested whether *MiMsp40* could suppress the callose response to elf18, a polypeptide consisting of the first 18 amino acids at the N-terminus of the bacterial elongation factor Tu (EF-Tu) protein, which can elicit an innate immune response in plants^44^. We inoculated *MiMsp40*-overexpressing and WT *A. thaliana* lines with 1 μM elf18, stained the samples with aniline blue at 24 h post-inoculation (hpi) to visualize the callose, and quantified the results using Quantity One (Bio-Rad). *MiMsp40* lines exhibited up to 60% less callose production than WT lines (Fig. 6A,B). These results suggest that *MiMsp40* can interfere with *Arabidopsis* PTI against *M. incognita*.

### Expression analysis of plant defence genes

To further validate the PTI suppression response, *MiMsp40* overexpression and WT *A. thaliana* lines were treated with 1 μM elf18 to monitor changes in the expression of defence genes. The mRNA expression levels of *FRK1*, *PAD4*, *WRKY33*, *WRKY29* and *CYP81F2* were measured by qPCR; the expression of these genes has been shown to significantly increase plant defences and to work as PTI-responsive markers^18^,^45^. As expected, after the plants were treated with elf18 for 6 hours, all five marker genes were strongly induced in WT plants and were repressed in plants overproducing MiMsp40 (Fig. 6C). These data further confirm that *MiMsp40* can function as a suppressor of *Arabidopsis* PTI responses.

## Discussion

The *MiMsp40* gene was isolated approximately one decade ago as a clone from an *M. incognita* gland-specific cDNA library^4^; however, little is known about its roles in nematode-plant interactions. Although *Msp40* was highly conserved in exon/intron distribution and protein composition in five tested *Meloidogyne* species (Figs 1A and S1A), no putative orthologue was present in non-*Meloidogyne* PPNs or animal-parasitic or free-living nematodes for which genome or transcriptome sequences were available. The phylogenetic relationships among the five *Meloidogyne* species in the *Msp40* consensus tree corresponded with a phylogenetic tree based on other characteristics such as morphology, karyotype, and reproduction mode^38^. Although it could be inflated by the absence of global *Meloidogyne*-specific genes, this analysis may still provide an improved understanding of the evolution of nematode species or even intrasubspecific strains (Fig. S1B), along with a range of aspects of PPN parasitism biology^46^.

Under adverse conditions, such as resistant plant genotype or unfavourable host species, specific obstructive characteristics usually develop during the nematode-plant interaction and may be used as indicators of host suitability. Therefore, ectopic expression and engineering of RNAi of parasitism genes in host plants have been adopted broadly to gain initial insights into the functions of nematode effector proteins secreted during infection. To demonstrate the role of *MiMsp40* in plant parasitism, we produced transgenic *Arabidopsis* overexpressing *MiMsp40* and evaluated the subsequent effect on nematode parasitism. Although both *MiMsp40* overexpression and WT lines developed mature females, a significant increase in the number of knots and eggs on independent transgenic lines occurred compared to the WT lines. Conversely, plant host-derived *MiMsp40* RNAi did not induce morphological differences but reduced nematode susceptibility significantly compared with the WT lines. These results are partly consistent with observations with other PPN effectors such as *16D10*^42^, *Mi-CRT*^33^, *Mi8D05*^47^, and *GrUBCEP12*^30^ and provide strong evidence that *MiMsp40* is involved in plant parasitism. Additionally, *MiMsp40* overexpression promoted plant root growth even though the difference in growth was small, suggesting that this protein may be involved in diverse pathways in the host.

Although the results of analysis of *in planta* immunolocalization for MiMsp40 were negative and thus could not directly confirm its secretion and localization in host tissue, several lines of evidence suggest that MiMsp40 is secreted during parasitism. First, *in situ* hybridization indicated exclusive expression in *M. incognita* secretory esophageal gland cells. Second, the presence of a typical N-terminal signal peptide in the protein sequence could target it to the secretory pathway. Third, *MiMsp40* mRNA intensively increased at the par-J2 stage and then gradually declined at later stages (Fig. 1C); this developmental expression pattern of *MiMsp40* suggests a role in pathogenesis most likely during migration and the establishment of nematode feeding sites. Finally, similar to some typical nematode effectors, *Msp40* transgenic expression affects plant development and susceptibility to root-knot nematodes compared with the WT line, indicating that Msp40 can function *in planta* in a manner conducive to successful parasitism. Therefore, the combined results establish that Msp40 is a secreted protein involved in parasitism.

Elucidation of the subcellular localization of MiMsp40 *in planta* could help to determine the locations at which it performs its function and to identify potential host targets. Theoretically, the N-terminal signal peptide is cleaved off, and the mature protein is delivered into the host cell through the nematode stylet. However, considering the functional diversity among the signal peptides of different effectors^40^,^48^, we investigated where the MiMsp40 effector would localize and whether its signal peptide has transmembrane transport activity in plant cells. Consistent with the transient expression of *eGFP*, both constructs of *MiMsp40* coding sequences (remaining or minus signal peptides) fused to eGFP reporter genes under the control of the *CaMV 35S* promoter and exhibited GFP signals within the cytoplasm (Fig. S2), providing evidence for cytoplasmic localization but not extracellular or nuclear targeting. In addition, the MiMsp40 signal peptide did not function properly in plants coordinated with *HsCBP*^40^; the opposite was noted for *Gr-CLE*^48^. Therefore, the discrepancy in the functions of nematode native signal peptides in plant cells may be associated with their sequence diversity.

In plants, the initiation of PCD in response to invading pathogens is frequently associated with resistance to subsequent pathogen multiplication and spread. Because animal and plant cell death programs share molecular mechanisms, the pro-apoptotic mouse protein Bax can induce plant PCD that physiologically resembles the cell death associated with defence-related HR^49^. The suppression of PCD is a potentially important virulence mechanism for biotrophic pathogens that extract nutrients from living plant cells. As a result, the ability to suppress BT-PCD is a valuable initial screen for pathogen effectors capable of suppressing defence-associated PCD^50^,^51^. In our infiltration assay, MiMsp40 consistently suppressed BT-PCD, whereas infiltration with GFP before Bax or Bax alone simultaneously initiated a typical BT-PCD reaction (Fig. 3A), thereby suggesting that MiMsp40 might suppress cell death by being a modular effector targeting a step of the common PCD pathways.

According to the ‘zig-zag’ model, pathogens evolved effectors with PTI or ETI modulation activity during pathogen-plant interactions. To determine the type of immunity suppressed by MiMsp40, we inoculated *MiMsp40*-overexpressing and WT *A. thaliana* lines with the bacterial PTI elicitor elf18 and confirmed PTI inhibition: (I) compared with WT plants, *MiMsp40* plants exhibited a significant decrease in the production of callose, a well-studied PTI component associated with cell wall defence (Fig. 6A); and (II) a batch of defence-related genes that play diverse roles in PTI responses and are commonly considered essential markers displayed reduced mRNA levels during *MiMsp40* expression (Fig. 6C). Both results were similar to the effects of plant ectopic expression of *Mi-CRT*^33^, suggesting that MiMsp40 can interfere with the *Arabidopsis* PTI response against *M. incognita*.

Moreover, our co-agroinfiltration assays showed that MiMsp40 suppressed MKK1- or NPK1-triggered pathogen-independent cell death in *N. benthamiana*. Therefore, this protein is a potent suppressor of macroscopic cell death triggered by MAPK cascades and specifically targets components downstream of MKK1 and NPK1. Plant MAPK cascades play pivotal roles in signalling plant defences against pathogen attacks and are considered crucial for PTI and ETI responses.

We then determined whether MiMsp40 also suppressed ETI-associated cell death. Select effectors appear likely to suppress ETI triggered by other effectors. Therefore, we determined whether a similar effect was noted following the transient co-expression of MiMsp40 combined with ETI inducers (the cytoplasmic NBS-LRRs R3a and Gpa2 and their cognate elicitors Avr3a and RBP1, respectively) in *N. benthamiana* leaves. As a result, MiMsp40 suppressed PCD triggered by R3a/Avr3a but not by Gpa2/RBP-1. In contrast, GrCEP12 suppressed cell death triggered by both groups of cognate elicitors^30^. Therefore, MiMsp40 is a novel nematode-secreted ETI suppressor that may target key component(s) involved in R3a/Avr3a (but not Gpa2/RBP-1)-induced immune signalling.

Extensive overlap and spatial interplay between ETI and PTI signalling pathways have been noted in other pathogen-plant systems^52^, and numerous effectors with dual suppression activity have been confirmed in plant pathogenic bacteria^34^, fungi^35^,^36^ and oomycetes^37^ with common targets and multiple activities. Therefore, Msp40 may suppress PTI and ETI to prevent PCD events by targeting common component(s) (such as elements of MAPK cascades) or by interacting with diverse components of two immunity pathways.

Specific immunomoderators of PPN, such as calreticulin (Mi-CRT), cellulose binding protein (HsCBP), Venom Allergen-like Protein (GrVAP1), and annexin-like protein (HS4F01), are homologous in several PPN species^53^, suggesting that they reflect early evolutionary adaptations to mitigate basal host defences during host invasion and migration. However, Msp40 may have evolved later because of the absence of homologous sequences in other organisms. The advantageous expression of this protein in the subventral oesophageal gland during the parasitic J2 stage indicates that root-knot nematodes secrete Msp40 into the plant cytoplasm to suppress host defences primarily during the early parasitic stages of invasion, migration and feeding cell formation. This process is favourable for the *Meloidogyne*-specific parasitic lifestyle and/or an extensive host range. Further studies such as screening of host target(s) and identification of pathologic cascades are required to elucidate the role of *Meloidogyne*-secreted Msp40 in plant parasitism.

## Materials and Methods

### Plant materials and growth conditions

Seeds of *A. thaliana* ecotype Col-0 were surface-sterilized for 20 min in 2% (w/v) sodium hypochlorite and subsequently washed three times with sterile water. The plants were grown under sterile conditions on Murashige and Skoog (MS) solidified medium containing 2% sucrose or in potting soil in a growth chamber under long-day conditions (16 h light/8 h dark) at 23 °C and 70% relative humidity.

### Nematode culture

*M. incognita* strains were propagated in axenic cultures starting from a single-egg mass on the roots of greenhouse-grown tomato plants (*Solanum lycopersicum* var. ‘Baiguo’). Egg masses on root galls were handpicked and hatched in water at 25 °C in improved Baermann pans for 48–72 h, and the hatched preparasitic second-stage juveniles (pre-J2s) were collected for use in root infection assays or nucleic acid extractions. Mixed parasitic stages of *M. incognita* were collected from infected tomato roots by root blending and sieving as previously described^54^. Parasitic J2 (para-J2), J3 and adult female nematode stages were separated under a microscope and used for quantitative reverse-transcription polymerase chain reaction (qRT-PCR) analysis.

### DNA and RNA isolation

For nematode genomic DNA isolation, *M. incognita* pre-J2s were ground into powder in liquid nitrogen and incubated in SDS lysis buffer (100 mM NaCl, 100 mM Tris-HCl [pH 8.5], 50 mM EDTA [pH 7.4], 1% SDS, 1% β-mercaptoethanol, and 100 μg/ml proteinase K) at 65 °C for 60 min. The samples were then sequentially extracted with phenol/chloroform (24:1), precipitated with ethanol, and treated with RNase. The purified DNA was resuspended in double-distilled water and diluted to 1 μg/μl stocks at −20 °C. Both nematode and plant total RNA was isolated using TRIzol RNA extraction reagent (Invitrogen, USA) following the manufacturer’s instructions, and the DNase-treated RNA was store at −80 °C.

### Sequence analyses

The *M. incognita Msp40* sequence (Acc no. AY422833) was used to design primers^4^. The cDNA and genomic DNA of this sequence were cloned by PCR amplification. To screen for homologies, the *MiMsp40* cDNA sequence was searched using BLAST against the genome and transcriptome databases for *H. glycines*, *G. rostochiensis*, *G. pallida*, *B. xylophilus*, and other plant-parasitic and free-living nematodes; the derived sequences were downloaded. *M. javanica* and *M. arenaria Msp40*-like sequences were obtained by PCR using the full-length gene primers *Msp40cds* F/R (Table S1) and a corresponding genomic DNA template. A comparison of the nucleotide and predicted amino acid sequences of the retrieved *MiMsp40* homologies was conducted using Clustal X 2.0, and the predictions of the secretory signal peptide and conserved domain were performed using SignalP 4.1 (http://www.cbs.dtu.dk/services/SignalP) and an NCBI CD-Search (http://www.ncbi.nlm.nih.gov/Structure/cdd/wrpsb.cgi). The *in planta* subcellular localization of the MiMsp40 mature protein was predicted using Psort software (http://psort.hgc.jp/form.html). The percentage similarities of five Msp40-like proteins for the corresponding nucleotide sequences were calculated using MEGA 5 software, and a phylogenetic analysis was performed using the neighbour-joining (NJ) method to demonstrate the phylogenetic relationship among the five *Meloidogyne* species.

### DNA gel blot analysis

In total, 10 μg of genomic DNA (*M. incognita* strains ‘TS’ and ‘SY’) was digested overnight at 37 °C with *EcoR*I and *BamH*I restriction enzymes (New England Biolabs), separated by 0.7% agarose gel electrophoresis, and transferred onto an Hybond N^ + ^membrane (GE Healthcare) by a capillary process. The Southern probe was generated using a full-length *MiMsp40* cDNA template with specific primers M40F/R (Table S2) and a PCR DIG Probe Synthesis Kit II (Roche Applied Science, Indianapolis, IN, USA). Probe labelling, hybridization and signal detection were performed following the kit’s protocol.

### *In situ* hybridization

To evaluate the spatial distribution of *MiMsp40* expression, parasitic stage J2s were fixed as previously described^55^. The primers M40F and M40R for *MiMsp40* cDNA were used to synthesize the DIG-labelled sense (negative control) and antisense cDNA probes by asymmetric PCR^56^. Hybridization was performed following de Boer’s method^55^. Hybridization signals within the nematodes were detected with alkaline phosphatase-conjugated anti-digoxigenin antibody and substrate, and specimens were observed under a light microscope.

### Quantitative RT-PCR (qRT-PCR) analysis of RKN and *Arabidopsis* genes

DNase-treated RNA (1 μg) was used for cDNA synthesis and PCR amplification using the SuperScript® III First-Strand Synthesis System (Invitrogen) according to the manufacturer’s protocol. *M. incognita* gene-specific primers for *Msp40* and *α-Tubulin*, as well as *Arabidopsis* gene-specific primers for *FRK1* (flg22-induced receptor-like kinase 1), *PAD4 (*phytoalexin deficient 4), *WRKY29*, *WRKY33*, *CYP81F2* (cytochrome p450, family 81, subfamily f, polypeptide 2), and *UBP22* (ubiquitin-specific protease 22), were designed (Supplemental Table S2). Each 25 μl reaction mixture was prepared using SYBR Premix Ex Taq™ (TaKaRa, Japan) according to the manufacturer’s protocol, and the PCRs were run on an ABI7500 Real-Time PCR System (Applied Biosystems) using the following program: 95 °C for 5 min followed by 40 cycles of 95 °C for 30 s and 60 °C for 30 s. For all the tested genes, at least three independent experiments with three technical replicates of each reaction were performed. *A. thaliana UBP22* and *M. incognita α-Tubulin* were employed as internal controls to normalize the gene expression levels (Table S2). The results were analysed using SDS 2.0 software (Applied BioSystems), and gene expression changes were calculated using the 2^−△△CT^ method^57^.

### Subcellular localization

The *MiMsp40* gene with and without signal peptide-encoding regions was amplified using the gene-specific primer pairs M40-F1 *Kpn*I/M40- R1 *Xba*I and M40-F2 *Kpn*I/M40- R1 *Xba*I, containing *Kpn*I and *Xba*I restriction enzyme sites in the forward and reverse primers, respectively (Supplemental Table S2). The resulting amplified fragments were cloned into the respective sites in a modified p35SeGFP vector between the cauliflower mosaic virus (CaMV) 35S promoter and enhanced green fluorescent protein (eGFP) to express the eGFP fusion protein; the p35SeGFP vector without *MiMsp40* was used as a control. Both constructs were confirmed by DNA sequencing. The resulting constructs were introduced into onion (*Allium cepa*) epidermal cells by biolistic bombardment with a PDS1000/He system (Biolistic Particle Delivery System, Bio-Rad, CA, USA). Onion pieces were incubated for 24 h at 24 °C in the dark; epidermal peels were then observed through a fluorescence microscope (Nikon Eclipse TE300, Tokyo, Japan).

### Plasmid construction and transgenic *Arabidopsis* plant generation

For host expression, the *MiMsp40* coding cDNA sequence (without the signal peptide region) was generated through amplification and subcloned into the *Nco*I/*BstE*II restriction sites of the vector pCAMBIA3301. The resulting vector p3301:Msp40 contained the coding sequence of *MiMsp40* in the sense orientation downstream of the CaMV35S promoter and a phosphinothricin (PPT) resistance gene, *bar*.

Two fragments of the *MiMsp40* coding region (336 bp and 293 bp) were selected for engineering the host-derived RNAi. The sense and anti-sense cDNA sequences were amplified from the full-length cDNA clone with gene-specific primers (Table S2) that introduced *Bgl*II, *EcoR*I, *Sal*I, or *BamH*I restriction sites. These amplified sequences were cloned into the *Bgl*II-*EcoR*I sites and the *Sal*I-*BamH*I sites of the pSAT4 RNAi vector^58^ to generate pSAT:TS1 RNAi and pSAT:TS2 RNAi, respectively. Both RNAi sequences were confirmed by PCR, endonuclease digestion and sequencing. These sequences were then subcloned as *Bgl*II/*BstE*II fragments into the binary vector pCAMBIA3301 to produce highly effective intron-containing RNA silencing constructs (p3301:TS1 RNAi and p3301:TS2 RNAi).

Both overexpression and RNAi constructs were introduced into the *A. tumefaciens* strain LBA4404 by the freeze-thaw method, and the transformations were confirmed by PCR. *A. thaliana* ecotype Col-0 plants were transformed by the floral dip method. Seeds were collected and germinated in soil. Transformants were selected by spraying phosphinothricin (PPT) at 100 mg/L 6 days after germination. The spraying was repeated twice. Homozygous T3 seeds were collected from T2 lines after a segregation analysis of PPT resistance and were used in this study.

### Nematode infection assays

The hatched *M. incognita* J2s were collected and surface-sterilized by incubating for 10 min in 0.004% mercuric chloride, 0.004% sodium azide, and 0.002% Triton X-100. The samples were then washed four times with sterile distilled water. The sterilized J2s were resuspended in low melting point agarose at a concentration of 100 J2s/10 μL and were inoculated onto 14-day-old *Arabidopsis* grown on MS medium at approximately 50 J2s per root tip. Infected seedlings were maintained under the identical conditions described above. Six weeks after inoculation, the numbers of knots and eggs were counted for at least 30 plants of each line, the average numbers were calculated, and statistically significant differences between transgenic lines and the wild-type controls were determined by *t*-test using the statistical package SPSS.

### Suppression of immune-associated programmed cell death

The suppression of PCD in *N. benthamiana* leaves was assessed as previously described^59^. The Msp40- and GFP-coded sequences were amplified and inserted into the *SmaI/SalI* sites of the PVX vector pGR107^60^. pGR107:NbMKK1 and pGR107:NbNPK1^Nt^ (residues 1 to 373) expression constructs were generated in a previous study^27^. The confirmed constructs were then introduced into the *A. tumefaciens* strain GV3101 by electroporation. The cultured *A. tumefaciens* cells (OD_600_ = 0.4) carrying *MiMsp40* and *GFP* were initially infiltrated into the leaves of *N. benthamiana* plants, which were grown in the greenhouse for 4–6 weeks at 25–28 °C under 16 h light/8 h dark. The identical infiltration site was then challenged with *A. tumefaciens* cells carrying the *Bax*, *MKK1* or *NPK1*^*Nt*^ gene at 24 h after initial inoculation. Simultaneously, *A. tumefaciens* strains carrying *Bax*, MKK1, NPK1^Nt^, *MiMsp40* or *GFP* genes were infiltrated alone as controls. The plants were monitored for symptoms, images were acquired 5 to 7 days after the last infiltration, and each infiltration area was then scored^61^. The experiment was repeated at least three times, and each assay consisted of at least five plants with three leaves (fifteen leaves total) inoculated similarly.

To examine whether MiMsp40 suppresses the hypersensitive response from ETI triggered by R proteins and their cognate elicitors, the coding fragment was cloned into pMD1 to generate the expression construct pMD1:MiMsp40. pMD1:GrCEP12 and the empty pMD1 plasmid were used as the positive and negative controls, respectively^30^. These constructs were transformed into the *A. tumefaciens* strain GV3101. The transient co-expression of R3a/Avr3a or Gpa2/RBP-1 was performed as described previously^30^. The symptoms were monitored, and images were acquired as described above.

### Western blot analysis

*N. benthamiana* leaves were harvested 4 days post infiltration. Protein extracts were prepared by grinding 400 mg of leaf tissue and incubating the sample in extraction buffer (50 mM Tris [pH 8.0], 2 mM EDTA, 2 mM DTT, 0.25 M sucrose and protease inhibitor). The supernatant was collected after centrifugation and separated on a 12% polyacrylamide gel. The proteins were transferred onto a polyvinylidene difluoride (PVDF) membrane using a semi-dry transfer system (BioRad). The membrane was then blocked in 5% skim milk (in PBS, pH 7.2) for 1 hour, incubated with primary antibody diluted with PBS (pH 7.2) containing 1% skim milk for 1 hour, washed with PBS-T (0.05% Tween-20 in PBS, 5 minutes × 3 times), incubated with HRP tag-conjugated secondary antibody diluted with 1% skim milk (in PBS, pH 7.2) for 1 hour, then washed again and detected using a chemiluminescence method.

### Callose staining and microscopic analysis

Leaves from 5-week-old *Arabidopsis* plants of *MiMsp40* and Col-0 lines were inoculated with 1 μM elf18. Samples inoculated with buffer were used as a negative control. Leaves were sampled after overnight incubation at room temperature, cleared with 100% methanol, and washed with sterilized water. After staining with aniline blue (0.05% in phosphate buffer, pH 8.0) for 24 h, leaf samples were examined under a photomicroscope, and the images were analysed using Photoshop software (Adobe). This experiment utilized three independent biological replicates.

## Additional Information

**How to cite this article**: Niu, J. *et al*. Msp40 effector of root-knot nematode manipulates plant immunity to facilitate parasitism. *Sci. Rep.* **6**, 19443; doi: 10.1038/srep19443 (2016).

## Supplementary Material

Supplementary Information
