# Supplementary material for: Msp40 effector of root-knot nematode manipulates plant immunity to facilitate parasitism
Source: Sci Rep. 2016 Jan 22;6:19443. doi: 10.1038/srep19443 (PMC4726423; doi:10.1038/srep19443)

Supplementary information

Msp40 effector of root-knot nematode manipulates plant immunity to facilitate parasitism

Junhai Niu[1, 2#], Pei Liu[1#], Qian Liu[1], Changlong Chen[1], Quanxin Guo[1], Junmei Yin[2,], Guangsui Yang[2], Heng Jian[1*]

1. Department of Plant Pathology and Key Laboratory of Plant Pathology of Ministry of Agriculture, China Agricultural University, Beijing 100193, China

2. Tropical Crops Genetic Resources Institute, CATAS / Key Laboratory of Crop Gene Resources and Germplasm Enhancement in Southern China, Danzhou, Hainan 571737, China

#: Both authors contributed equally.

*: Corresponding author

Prof. Heng Jian

Department of Plant Pathology, China Agricultural University, Beijing 100193, P. R. China

Phone and Fax: 86-10-62731102

E-mail: hengjian@cau.edu.cn

Supplemental Table S1.

The platforms about nematodes genome and transcripts used for *Msp40* homologies screening.

**Species**	**Types**	**Blast server links**
M. incognita	Root-knot nematode	http://meloidogyne.toulouse.inra.fr/blast/blast.html
M. hapla	Root-knot nematode	http://www.pngg.org/cbnp/index.php?option=com_wrapper&Itemid=8
M. floridensis	Root-knot nematode	http://nematodes.org/genomes/meloidogyne_floridensis/
G. rostochiensis	Potato cyst nematodes	http://nematode.net/NN3_frontpage.cgi?navbar_selection=speciestable &subnav_selection=Globodera_rostochiensis
G. pallida	Potato cyst nematode	http://www.sanger.ac.uk/cgi-bin/blast/submitblast/g_pallida
H. glycines	Soybean cyst nematode	http://nematode.net/NN3_frontpage.cgi?navbar_selection=speciestable &subnav_selection=Heterodera_glycines
B. xylophilus	Pine wood nematode	http://www.genedb.org/blast/submitblast/GeneDB_Bxylophilus
C. elegans	Free-living nematode	http://www.wormbase.org/tools/blast_blat
Other nematode species		http://blast.ncbi.nlm.nih.gov/Blast.cgi

Supplemental Table S2. Overview of the reference and target genes used in this study. (The underlined regions represent cleavages sites of restriction enzymes.)

Genes	Primers	Primer sequences (5′–3′)	Length	Usage
Msp40 (AY422833)	Msp40 cds F	GGTCATTCTTATAACTAAAAACCTTCAAAC	1150bp	Full length cDNA and DNA cloning
	Msp40 cds R	AATTACGAGAAACACGGATGAATAGAT		
	M40 qRT-F1<br>M40 qRT-R1	GCAAAGAATCATGTAATGGGGATACTG<br>TGAGCACAATCAGAAATCATAGTCGGT	244bp	qRT-PCR
	M40 Sb F<br>M40 Sb R	CCATTATTGCTTTTGCTTACCCTT<br>GCACAATCAGAAATCATAGTCGGTT	857bp	Southern blotting
	M40-- F1 KpnI<br>M40--F2 KpnI<br>M40-- R1 XbaI	CGG***GGTACC***ATGGAGGAGGATAAGGAAG<br>CGG***GGTACC***ATGGAGGAGGATAAGGGAGA<br>TGC***TCTAGA***AGCAGCAGCTGCTGCAAGT	945bp<br>882bp	Subcellular localization
	Msp40 P1S	AAGATGCTGAGAGTGCAGAGGAG	356bp	in situ hybridization
	MSP40 P1A	CTTGTCATCTTCCTCCTTTTTATCC		
	M40-NcoI	TTG**CCATGG**ATGGAGGAGGATAAGGAAGACAT	948bp	Host expression
	M40-BstE Ⅱ	TTT**GGTGACC**TTAAGCAGCAGCTGCTG		
	TS1-BglII	CC**AGATCT**AAGATGCTGAGAGTGCAGAGGAG	354bp	In vivo RNAi 1
	TS1-EcoRI	CC**GAATTC** CTTGTCATCTTCCTCCTTTTTATCC		
	TS1- SalI	CC**CTGCAGGTCGAC**CTTGTCATCTTCC		
	TS1-BamHI-BstEⅡ	AATT**GGATCCGGTGACC**AAGATGCTGAGAGTGC		
	TS2 -BglII	TTG**AGATCT**CATGTAATGGGGATACTGC	292bp	In vivo RNAi 2
	TS2- EcoRI	TTG**GAATTC**AGCTGCTGCAAGTCTCTT		
	TS2 -SalI	AA**GTCGAC**AGCTGCTGCAAGTCTCTT		
	TS2-BamHI-BstEII	CGC**GGATCCGGTTACC**CATGTAATGGGGATACTGC		
	Msp40_SmaI	TAT**CCCGGG**ATGGAGGAGGATAAGGGAG	882bp	Suppression of PCD
	Msp40_SalI	CTA**GTCGAC**TTA AGCAGCAGCTGCTGCA		
α-Tublin (CK983765)	Mi Tub qRT-F	AAAAGAGGCTGAGGGTTGTGATTG	206bp	qRT-PCR (Control)
	Mi Tub qRT-R	GAACAGAAAGAGTTGCGTTGTAGGG		
CHAS intron (EU049859)	CHAS F	CGTTTTATCTTAATTGGCTCTTC	1312bp	RT-PCR
	CHAS R	CCTGCAAATTGACCAAAAAAG		
FRK1 (AT2G19190)	FRK1 qRT_F	TGCAGCGCAAGGACTAGAG	108bp	qRT-PCR
	FRK1 qRT_R	ATCTTCGCTTGGAGCTTCTC		
PAD4 (AT3G5243)	PAD4 qRT_F	GCCGCTTTCACCGCACTTTGG	102bp	qRT-PCR
	PAD4 qRT_R	GAGAGATTGGTTTCCGAGCAGAGG		
WRKY29 (AT4G23550)	WRKY29 qRT_F	ATCCAACGGATCAAGAGCTG	120bp	qRT-PCR
	WRKY29 qRT_R	GCGTCCGACAACAGATTCTC		
WRKY33 (AT2G38470)	WRKY33 qRT_F	GCTGCTATTGCTGGTCACTCC	201bp	qRT-PCR
	WRKY33 qRT_R	GGTCTCCTCGTTTGGTTCTTCC		
CYP81F2 (At5g57220)	CYP81F2 qRT_F	GTGAAAGCACTAGGCGAAGC	183bp	qRT-PCR
	CYP81F2 qRT_R	ATCCGTTCCAGCTAGCATCA		
UBP22 (AT5G10790)	UBP22 qRT_F	GCCAAAGCTGTGGAGAAAAG	159bp	qRT-PCR (control)
	UBP22 qRT_R	TGTTTAGGCGGAACGGATAC		

Supplemental Fig S1. Alignment analysis for *Msp40* homologies. (A) Genomic structure of the *Msp40* homologies in five *Meloidogyne* species, exons are represented by black boxes and introns by a line. (B) Phylogenetic tree constructed by the neighbor-joining method, based on the derivated Msp40-like proteins from five *Meloidogyne* species. The three Msp40-based clades corresponding to the divergence for reproduction modes and cytogenetical karyotypes among the tested species. "#", data referenced from the previous description mainly (Castagnone-Sereno, 2006), *M. floridensis* belongs to diploid with only one karyotype (2n=36) (David et al., 2013), and other *Meloidogyne* species contained diverse chromosome numbers and aneuploid. The triploid and tetraploid were putative karyotypes.

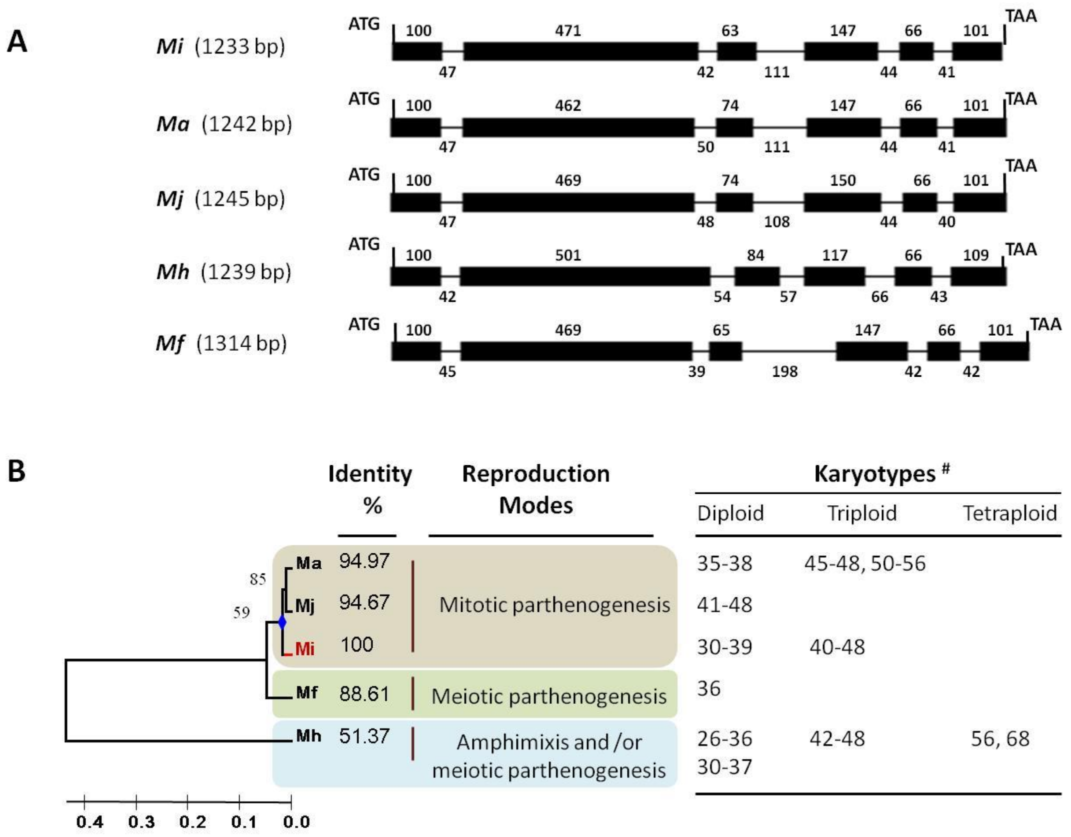

Supplemental Fig S2. Subcellular localization of MiMsp40 in plant cells. *MiMsp40* cDNA with and without signal peptide-coding sequence were fused to the joined GFP reporter gene respectively, and expressed in onion epidermal cells by particle bombardment. GFP fluorescence is mainly localized in the cytoplasm and the nucleus for both constructs.

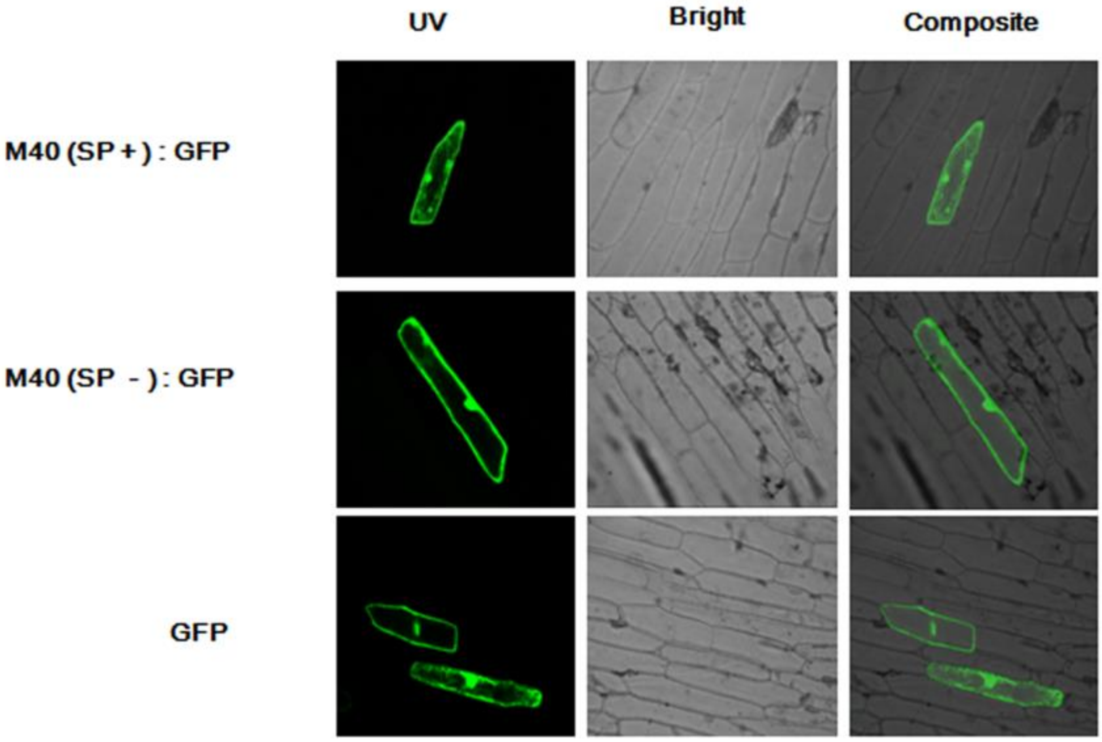

Supplemental Fig S3. Effect of plant host-derived RNAi of *MiMsp40* on *M. incognita* infection. (A) Root galls development of control (WT) and transgenic lines (TS1, TS2) of two different *MiMsp40* dsRNA cassette (TS1 RNAi and TS2 RNAi). (B) RT-PCR of the single-stranded CHAS intron of *MiMsp40* dsRNA hairpins confirmed the expression of TS1 and TS2 RNAi constructs in independent transgenic *Arabidopsis* lines.

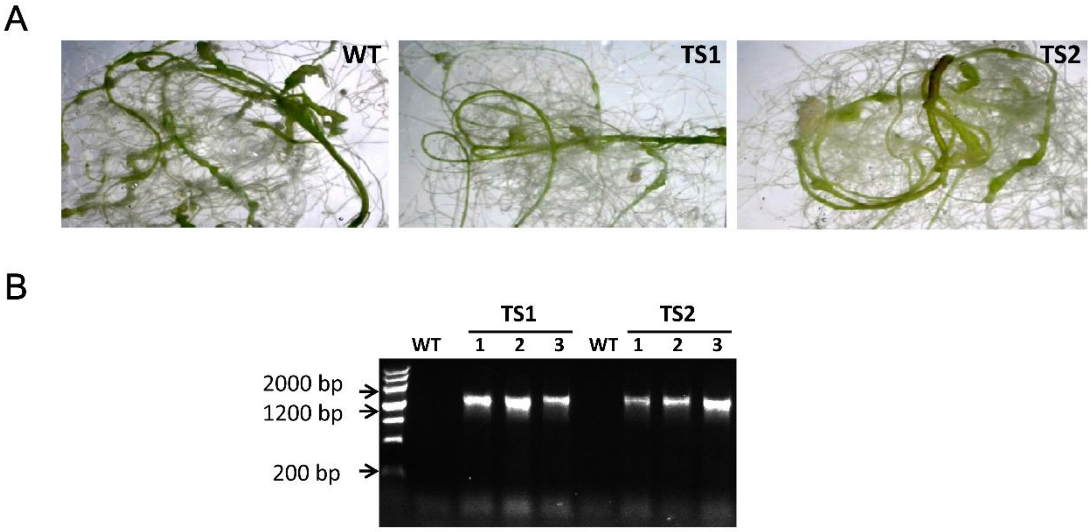

Supplement: Supplementary Information [file srep19443-s1.pdf]
